# Caliper measurement to improve clinical assessment of palpable neck lumps

**DOI:** 10.1308/003588412X13171221499784

**Published:** 2012-05

**Authors:** J Wasson, K Amonoo-Kuofi, J Scrivens, A Pfleiderer

**Affiliations:** Peterborough and Stamford Hospitals NHS Foundation TrustUK

**Keywords:** Head and neck neoplasm, Organ size, Physical examination, Ultrasonography

## Abstract

**INTRODUCTION:**

One-stop neck lump clinics with ultrasonography and cytopathology support are an expensive and finite resource. Consequently, many neck lump patients are assessed in general ear, nose and throat or head and neck clinics.

Optimal clinical assessment of neck lump size is important to guide investigation, monitor change and provisionally stage nodal disease. The aims of this study were to investigate whether caliper measurement is more accurate than clinical palpation in assessing neck lump size and whether caliper measurement of neck lump size correlates closely with accurate ultrasonography measurement.

**METHODS:**

A prospective study was carried out involving 50 patients with clinically palpable neck lumps presenting to the one-stop neck lump clinic. Long and short axis neck lump dimensions were estimated first by clinical palpation and second by caliper measurement. Estimations were compared with accurate ultrasonography measurement.

**RESULTS:**

The mean combined long and short axis measurement deviation from accurate ultrasonography measurement was smaller for caliper measurement (7.80mm) than for clinical palpation (12.38mm) *(p<*0.01). There was no significant difference observed between combined axis ultrasonography and combined axis caliper measurement of neck lumps (p=0.462).

**CONCLUSIONS:**

Caliper measurement is more accurate than clinical palpation in estimating the size of clinically palpable neck lumps. The use of calipers to measure the skin surface dimensions of palpable neck lumps is statistically comparable to accurate ultrasonography measurement.

Good clinical examination is an essential part of the initial assessment of neck lumps presenting to the ear, nose and throat (ENT) clinic. In 2004 the National Institute for Clinical Excellence (NICE) published guidance on improving outcomes in head and neck cancers.^1^ One of the published recommendations was the establishment of weekly rapid access one-stop neck lump clinics for patients presenting to their general practitioner (CP) with a suspicious neck mass. Ideally, such a clinic will have a clinician, cytologist and preferably a sonographer present to facilitate simultaneous clinical assessment, accurate fine needle aspiration (FNA) and ultrasonography of the patient’s neck. The purpose is to remove unnecessary steps from the patient care pathway and expedite diagnosis and necessary treatment. Sonography is important to guide FNA in small and deep neck lumps, provides information of lump morphology and can detect additional impalpable nodal enlargement, which facilitates provisional staging of suspected cancer.

In 2000 the Department of Health introduced waiting time targets for cancer.^2^ The two-week wait requirement for suspect cancer from GP referral to first hospital assessment can overburden the one-stop neck lump clinic, resulting in some overspill of suspect cancer patients into head and neck as well as general ENT clinics. While FNA of palpable superficial neck lumps can be performed in the absence of cytological support, assessment of neck lump size is usually an inaccurate subjective estimation. Accurate clinic assessment of neck lump size is important to help determine the need for further investigation, to monitor any growth of benign conditions and to provisionally stage nodal disease prior to definitive radiological staging.

A simple prospective study was performed in the one-stop neck lump clinic at Peterborough City Hospital with two aims: first, to investigate whether caliper measurement is more accurate than clinical palpation in assessing the two-dimensional size of neck lumps when compared with accurate ultrasonography measurement and, second, to investigate whether caliper measurement of neck lump size correlates closely with accurate ultrasonography measurement.

## Methods

**Figure 1:**
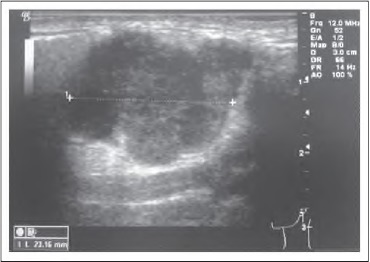
Ultrasonography measurement using a Philips iU22 xMATRIX ultrasonography machine

An eight-month prospective study was performed between January 2010 and August 2010. Data from 50 patients were collected from a weekly one-stop neck lump clinic that had both ultrasonography and cytopathology support. All patients presenting to the clinic with a visible and palpable neck lump requiring ultrasonography were included in this study. In each patient the presenting neck lump size was estimated in two dimensions: the longest and shortest axis. Three estimations of neck lump size were made and documented in sequence. First, the lump surface long and short axes were estimated by clinical palpation alone. Second, the same long and short axes were estimated using plastic calipers. Finally, definitive long and short axis dimensions were accurately measured using an iU22 xMATRIX (PhilipsHealthcare, Guildford,UK) ultrasonographymachine (Fig 1).

Care was taken to measure the ultrasonography lump image in a plane parallel to the skin surface so that the measurement was representative of the lump size palpable at the skin surface. This clinical-caliper-ultrasonography sequence of lump size assessment was strictly followed to ensure objective measurements did not influence subjective judgements. Five doctors (two consultants, two registrars and one staff grade doctor) participated in the study and one senior radiographer performed the sonography for all 50 patients. Measurement data were analysed and statistically tested using SPSS® vl7.0 (SPSS Inc, Chicago, IL, US).

## Results

### Caliper measurement vs clinical palpation

**Figure 2:**
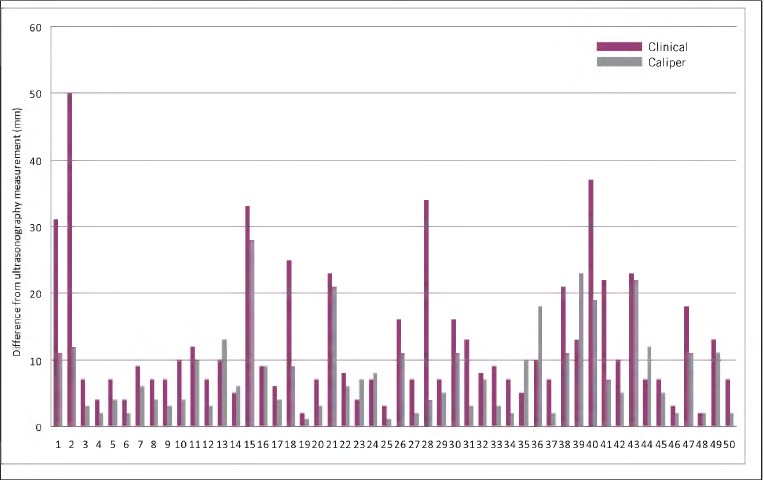
Combined long and short axis measurement deviation from ultrasonography measurement for clinical and caliper neck lump size assessment techniques for all 50 patients

To investigate whether caliper measurement is more accurate than clinical palpation in assessing two-dimensional neck lump size, the deviation in lump size estimation by both clinical and caliper techniques from accurate ultrasonography measurement was compared. For each technique, the difference between the estimated long and short axis measurements and the accurate ultrasonography long and short axis measurements was calculated. Both long and short axis deviations were then summated to obtain an aggregate deviation from the accurate ultrasonography measurement for both clinical and caliper techniques of neck lump size estimation. These summated deviated measurements for both techniques in all 50 neck lump patients are tabulated and illustrated in Figure 2. The mean aggregate deviation from ultrasonography measurement was smaller for caliper measurement (7.80mm) than for clinical assessment (12.58mm).

Both population samples (clinical difference and caliper difference) were tested for normality using a Shapiro-Wilk test. The p-values for both population samples were not significant for normality (pcO.Ol). A non-parametric Wilcoxon signed-rank test was therefore used to statistically test the differences between clinical and caliper measurements (Fig 2). A significant difference (pcO.Ol) was demonstrated between clinical and caliper techniques of neck lump size estimation when comparing summated measurement deviations from the accurate ultrasonography measurement of neck lump dimensions. The use of calipers to measure neck lump size in patients attending clinic was statistically more accurate than clinical palpation alone.

### Caliper measurement vs ultrasonography measurement

**Figure 3:**
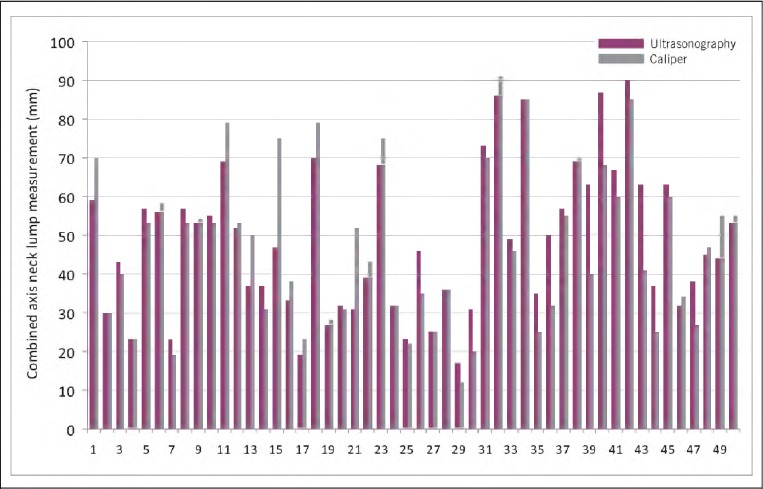
Combined long and short axis neck lump measurements for caliper and ultrasonography assessment techniques for all 50 patients

To investigate whether caliper measurement of neck lump size correlates closely with accurate ultrasonography measurement, long and short axis measurements were summated to derive a combined axis measurement for both caliper and ultrasonography assessment of each neck lump. Caliper and ultrasonography combined axis measurements for all 50 neck lumps are shown in Figure 3. The mean values for both these sample populations were similar: 47.26mm for combined axis caliper measurements and 48.26mm for combined axis ultrasonography measurements.

Both population samples (ultrasonography combined axis and caliper combined axis) were significant for normality (p>0.05) when applying the Shapiro-Wilk test and therefore statistically conformed to a normal distribution. A parametric, paired samples t-test was hence used to statistically test any significant difference between combined axis ultrasonography and combined axis caliper measurements of neck lumps (Fig 3). No significant difference (p=0.462) was observed between combined axis ultrasonography and combined axis caliper measurements of neck lumps. The use of calipers to measure the skin surface dimensions of palpable neck lumps was statistically comparable to accurate ultrasonography measurement.

## Discussion

Following their recommendation by NICE, one-stop neck lump clinics have been introduced across the National Health Service. Published audits have shown this initiative to be effective in expediting diagnosis and reducing steps in the patient care pathway.^3^, ^4^ However, there is a cost to providing this service with ultrasonography and cytopathology support. A one-stop service with FNA is estimated to cost £20,000 per clinic per year with an aggregate annual cost for England and Wales estimated at £2.2 million for 57 cancer service networks.^1^ This required expenditure probably explains why many ENT departments do not provide regular one-stop neck lump clinics and why ENT departments that do provide a one-stop service can become overwhelmed with two-week wait demands of suspected cancer referred by the GP. Consequently, many new and follow-up patients with neck lumps will be reviewed in either head and neck or general ENT clinics without ultrasonography or cytopathology support.

In these circumstances, accurate clinical estimation of lump size is important in the assessment and monitoring of neck lump patients and it can help determine the need for further investigation. For example, patients presenting to our unit with a palpable lymph node greater than 9mm will undergo FNA and possibly ultrasonography, suspicious metastatic nodal disease will require provisional clinical staging prior to definitive radiological staging, and benign head and neck conditions under follow-up may require serial measurement to identify any progressive mass enlargement. The use of an adjunct to improve size estimation will therefore enhance clinical examination and decision making.

Previous studies have shown ultrasonography to be an effective tool in assessing the accurate dimensions of head and neck tumours.^5^, ^6^ In this study, most patients presenting to the one-stop neck lump clinic did not require surgical resection of their presenting neck lump. Ultrasonography therefore provided an accurate method of lump measurement against which the less objective measurement techniques of clinical palpation and caliper measurement could be compared. A MEDLINE® literature search did not reveal any published studies that have compared clinical, caliper and ultrasonography techniques of lump size evaluation in the neck. This study is the first to show that simple caliper measurement of neck lump size is statistically more accurate than clinical palpation alone.

Furthermore, there is no statistically significant difference between caliper measurement and accurate ultrasonography measurement of lumps in the neck. While this close correlation between caliper and ultrasonography measurements has been demonstrated previously for lumps greater than 50mm in the breast,^7^ this is the first paper to identify that caliper measurement is statistically comparable to accurate but more costly ultrasonography of neck lump dimensions.

Ultrasonography is an excellent radiological tool for the investigation of neck lumps presenting to the clinic. It provides valuable information regarding morphology, precise anatomical origin, relation to important structures and vascularity of neck lumps. Moreover, ultrasonography can precisely identify impalpable neck pathology and accurately guides FNA of small and deep neck lumps, in addition to providing accurate three-dimensional measurements. Clearly, ultrasonography is always indicated when any presenting lump requires detailing or targeting and simple calipers will never substitute this sophisticated tool. This study simply highlights the ability of calipers to measure surface dimensions of palpable neck lumps accurately and to improve clinical assessment, particularly when an ultrasonography machine is not readily available.

Caliper measurement has its limitations. It is unable to provide three-dimensional measurements and accuracy is dependent on surface palpability of the presenting neck lump. Caliper surface measurement of neck lumps will therefore be compromised in deep structures such as the thyroid gland and in obese patients with considerable overlying adipose tissue. This difficulty was experienced by clinic doctors anecdotally but was not captured in the data.

## Conclusions

The calipers used in this study cost less than £1. We have shown that this inexpensive measurement tool is reliably accurate in assessing the dimensions of palpable neck lumps. For new and follow-up neck lump patients presenting to ENT clinics without ultrasonography and cytopathology support, we would advocate the use of calipers as an adjunct to help in the clinical assessment and management of their presenting neck lump. Furthermore, we advocate the use of calipers in the one-stop neck lump clinic for purposes of initial and serial measurements of palpable lumps, reserving ultrasonography to detail lump morphology, anatomy, impalpable or poorly palpable pathology and to guide precise FNA.

## Acknowledgement

We would like to thank Mr Nazir Bhat for permitting this study to be conducted in his one-stop neck lump clinic.

## References

[1] National Institute for Clinical Excellence (2004). Improving Outcomes in Head and Neck Cancers.

[2] Department of Health (2000). The NHS Cancer Plan.

[3] Ganguly A Giles TE Smith PAet ai. The benefits of on-site cytology with ultrasound-guided fine needle aspiration in a one-stop neck lump clinic Ann R Coii Surg Engl 2010 92 660 664 10.1308/003588410X12699663905032a PMC3229373 20663278

[4] (2000). Patients with neck lumps: can they be managed in a ‘one-stop’ clinic setting?. Clin Otolaryngol Allied Sci.

[5] (2006). Observation of tumour thickness and resection margin at surgical excision of primary oral squamous cell carcinoma – assessment by ultrasound. int J Oral MaxiHofac Surg.

[6] (2001). Clinical trial on the accuracy of a freehand and sensor-independent three-dimensional power Doppler ultrasound system measuring diameters, volumes and vascularity of malignant primaries of the neck. Ultraschall Med.

[7] (2004). Measurement of tumour size in case selection for breast cancer therapy by clinical assessment and ultrasound. EurJ Surg Oncol.

